# Synergetic Toxic Effect of an Explosive Material Mixture in Soil

**DOI:** 10.1007/s00128-013-1090-8

**Published:** 2013-09-05

**Authors:** Katarzyna Panz, Korneliusz Miksch, Tadeusz Sójka

**Affiliations:** Environmental Biotechnology Department, Silesian University of Technology, Akademicka 2, Gliwice, Poland

**Keywords:** Explosive materials, Phytotoxicity, Zootoxicity, Forest soil

## Abstract

Explosives materials are stable in soil and recalcitrant to biodegradation. Different authors report that TNT (2,4,6-trinitrotoluene), RDX (hexahydro-1,3,5-trinitro-1,3,5-triazine) and HMX (octahydro-1,3,5,7-tetranitro-1,3,5,7-tetrazocine) are toxic, but most investigations have been performed in artificial soil with individual substances. The aim of the presented research was to assess the toxicity of forest soil contaminated with these substances both individually as well in combinations of these substances. TNT was the most toxic substance. Although RDX and HMX did not have adverse effects on plants, these compounds did cause earthworm mortality, which has not been reported in earlier research. Synergistic effects of explosives mixture were observed.

Explosives are used on a large scale by both the military and by various civilian industries (e.g. mining, high-energy metalwork, and civil engineering) (Krishnan et al. 2000; Winfield et al. 2004; Vila et al. 2007a). Munitions manufacturing, transport, and utilization contribute to the high environmental contamination. 2,4,6-trinitrotoluene (TNT), hexahydro-1,3,5-trinitro-1,3,5-triazine (RDX, hexogen) and octahydro-1,3,5,7-tetranitro-1,3,5,7-tetrazocine (HMX, octogen) are found mainly in soils and surface waters; there have also been cases of groundwater contamination (Winfield et al. 2004). Most explosives are stable due to their chemical structure and potential for binding to the organic matter, thus making soil remediation difficult (Rylott et al. 2011). Additional limitation is the high toxicity of explosives (Krishnan et al. 2000).

Trinitrotoluene is highly toxic to terrestrial plants. In most cases the toxic effects (germination rate, decrease of plant biomass, and abnormal growth) are directly related to an increase TNT concentration (Krishnan et al. 2000; Vila et al. 2008). Hexogen, even in high concentrations, does not affect seed germination; however, many adverse developmental effects have been detected in plants exposed to this substance. Some of effects (e.g. atypical bilateral symmetry, bifurcated and fused leaves, irregular and curved leaf margins, and underdeveloped roots) were indicative of teratogenicity (Winfieled et al. 2004; Vila et al. 2007b). Octogen, even in very high concentrations, is not toxic to higher plants (Rocheleau et al. 2008, 2003).

Trinitrotoluene acute toxicity was observed in tests with earthworm *Eisenia andrei* (Lachance et al. 2004) and other oligochaete species (*Enchytraeus crypticus* and *Folsomia candida*; Schäfer and Achazi 1999). Concentrations causing 50 % mortality (LC_50_) (Best et al. 2006) ranged from 143 to 365 mg/kg and depended on soil type. Trinitrotoluene has also exhibited adverse effects on earthworm biomass and has shown to cause reduction in reproduction parameters (decrease in cocoon and juvenile numbers; Schäfer and Achazi 1999; Robidoux et al. 2002). Hexogen and octogen were not lethal to earthworms, but as with TNT, they caused biomass and fertility decrease (Robidoux et al. 2002; Simini et al. 2003).

Even though there is significant information about the toxicity of explosives, there are still a lack of reports about the influence of different kinds of soil related to their associates on toxic effects. Moreover, most of the tests conducted thus far have been based on determining toxic effects of individual substances. Yet in the environment, explosives usually appear as mixture, which can cause difficulty in assessing their individual effects, as can be observed with other groups of contaminants (Kalka 2012). The main purpose of this research was to assess the phyto- and zootoxicity of mixtures of explosives (and individual explosives) in forest soil.

## Materials and Methods

The soil used in this research originated from the Panewnickie Forests situated near Katowice (Poland). It was sandy soil (sand content >74.7 %), with low pH (3.8 ± 0.5) and organic carbon content (<5.24 %).

Explosives used to spike the soil were technical grade and in the crystal form. Prior to addition to the soil, they were dissolved in acetone. TNT, RDX, and HMX solutions were added to a small sample of the soil (100 g). Following acetone evaporation, additional soil was added to each sample. Nominal concentrations of explosives measuring 100, 180, 360, 540, and 1,000 mg/kg were identical in both tests. In the samples with explosives mixture (MIX), equal amounts of each compound were used to achieve identical concentrations, as in tests with individual substances (e.g. in the sample with 100 mg/kg nominal concentrations were 33.3 mg/kg TNT, 33.3 mg/kg RDX, and 33.3 mg/kg HMX). Unspiked soil was used for control samples.

Explosives concentrations in soil after spiking were measured according to US EPA Method 8330 (with the some modifications in the procedure). This method provides high performance liquid chromatographic conditions for the detection of explosives. Prior to using this method, extraction in an ultrasonic bath with the use of acetonitrile must be conducted. In this research, samples were sonicated for 2 h (instead of 18, which is recommended in Method 8330) and then shaken for 18 h (rpm = 100). Other stages of sample preparation were identical to US EPA Method 8330. The separation of explosives in liquid extracted samples was conducted with the use of Thermo Scientific, Hypersil Gold C18 250 × 4.6 mm chromatographic column (filling granulation – 5 μm) preceded with precolumn Hypersil Gold 10 × 4 mm (filling granulation – 5 μm). The mobile phase during the separation was 50/50 methanol/organic free reagent water. Flow rate through the column was 1 ml/min (lower than recommended by Method 8330, which is 1.5 ml/min); injection volume was 20 μl (in Method 8330 – 100 μl). Method validation was conducted with the use of analytical standard EPA 8330 MIX A produced by Supelco containing 8 explosives dissolved in acetonitrile. Explosives concentrations were measured in each sample type after spiking the soil and are presented in Table 1. Nominal concentrations are used in the figures and in the text to make results clear and easy to compare.

**Table 1:** Nominal and measured concentrations of explosives in soil used in toxicity tests

Explosive	Nominal concentration (mg/kg)	100	180	360	540	1,000
TNT	Measured concentration (mg/kg)	98.1	175.7	348.5	519.5	954.3
RDX		98.8	180.5	347.8	506	976
HMX		98.6	176.2	355	532.4	991.1
	Nominal concentration (mg/kg)	33.3	60	120	180	333.3
TNT in MIX	Measured concentration (mg/kg)	32	57.2	116.2	176.6	325.3
RDX in MIX		32.2	59	114.8	175.7	329.3
HMX in MIX		33.7	59.2	115.1	177.5	328.6

Phytotoxicity tests were conducted according to PN-ISO 11269-2:^2001^ “Effects of chemicals on the emergence and growth of higher plants.” One monocotyledonous plant – bread wheat (*Triticum aestivum*) – and one dicotyledonous plant – red clover (*Trifolium pratense*) – were chosen for the test. Only untreated seeds with germination ability greater than 90 % were used in the test. Twenty seeds were sowed in each pot (containing 500 g of soil). Soil was watered with distilled water; the soil moisture was established at the level of 40 %. Each sample was prepared in quadruplicates. The experiment was carried out in a plant growth chamber at the temperature of 21°C/18°C (day/night). Air humidity in the chamber was kept at the level of 80 %; light intensity was 25,000 lm/m^2^ surface in the hourly cycle of 14/10 (day/night). After 7 days following the beginning of the test, germinated seeds were counted in each sample. Subsequently, the 5 most representative seedlings in each sample were chosen; the rest were removed. After 14 days, plants were collected, lengths of shoots and roots were measured, and biomass of fresh shoots was weighed.

Acute earthworm toxicity tests were conducted on the base of PN-ISO 11268-1:^1997^ “Effects of chemicals on earthworm (*Eisenia fetida*).” Plastic pots were filled with 750 g of contaminated soil, which moisture was established at the level of 40 % using a manure and water solution (manure served as food for the earthworms). Control samples were unspiked and were comprised of soil with manure and water solutions. Samples were prepared in quadruplicates. Ten washed earthworms (100–600 mg weight) were introduced into each container; containers were covered with gauze to prevent the earthworms from escaping. Test was conducted for 14 days at the room temperature and in stable soil moisture. After 2 weeks, living organisms were counted (mortality assessment) and weighed (effect of chemicals on the biomass).

Toxicity endpoints such as LC_50_ were calculated on the basis of the linear regression best fitting model. Statistically significant differences between the results were evaluated on the basis of determining standard deviation and on the basis of Dunnett’s multiple comparison test (*p* ≤ 0.05).

## Results and Discussion

**Fig. 1:**
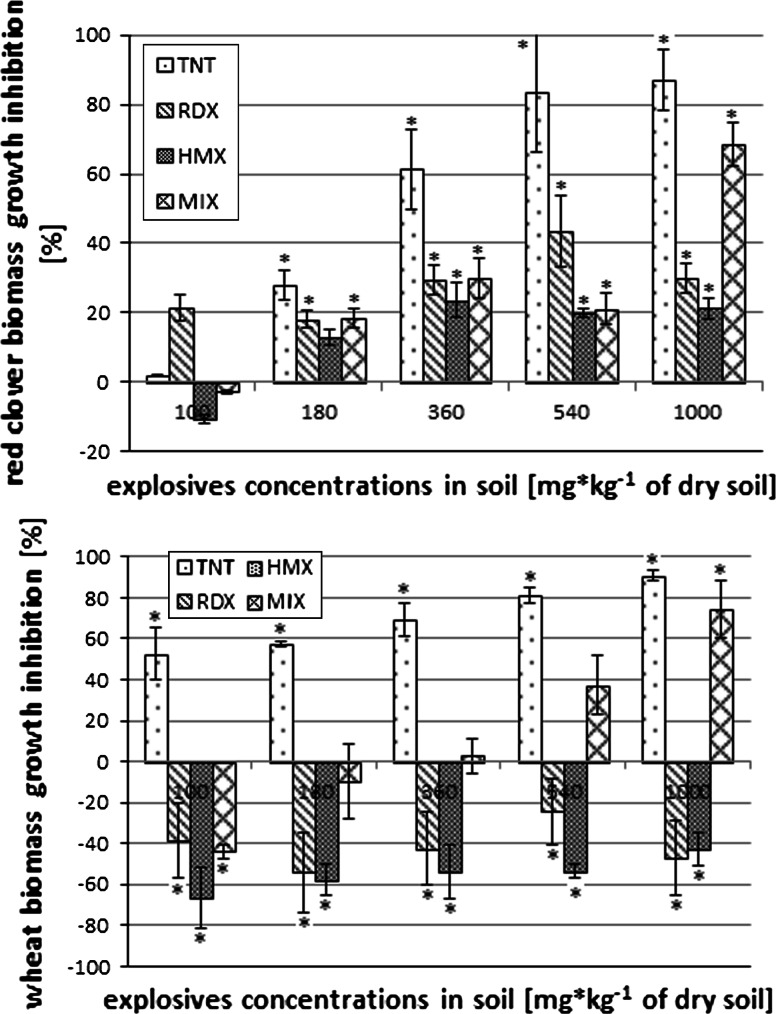
Effect of different explosives concentrations on the biomass of red clover and bread wheat weight (*statistically significant results, *p* ≤ 0.05)

In the phytotoxicity evaluation of soil contaminated with explosives, inhibition of seed germination, biomass weight, and root growth for two plants (*T. pretense* and *T.* *aestivum*) were determined. It has been stated that a concentration 180 mg/kg of each explosive (except HMX) and a mixture of explosives in soil cause significant biomass loss of red clover seedlings (Fig. 1). For wheat seedlings, the significant fresh biomass loss was observed in each TNT concentration, while HMX and RDX caused increased growth in comparison with the control samples (in each analyzed concentration). In samples with the explosives mixture, growth stimulation was noticed in lower concentrations, while in the concentrations of 360 mg/kg and higher significant (in comparison with control samples) plant biomass weight loss was observed (Fig. 1). In the samples spiked with TNT at the greatest concentrations (540 and 1,000 mg/kg), chlorosis on the red clover leaves surface was visible. In the last 5 days of the experiment drying and death of the seedlings were observed.

**Fig. 2:**
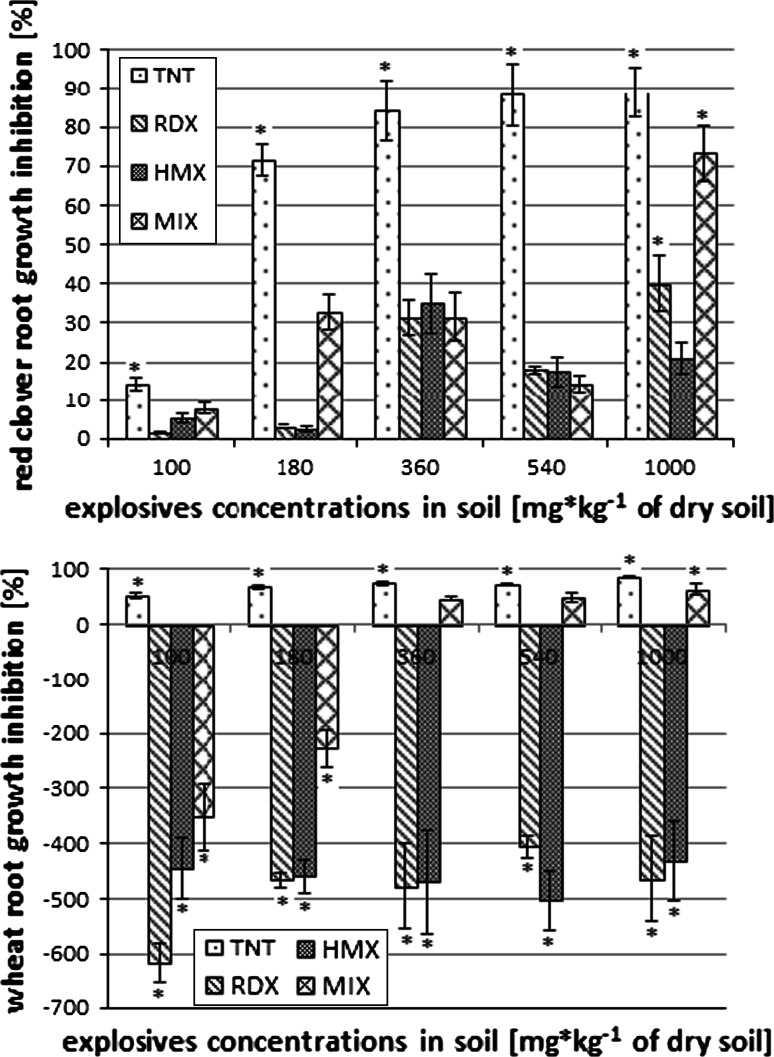
Effect of different explosives concentrations on the root length of red clover and bread wheat weight (*statistically significant results, *p* ≤ 0.05)

The analysis of root length demonstrated that 2,4,6-trinitrotoluene caused a significant decrease of root length in red clover and wheat in all applied concentrations. Hexogen and octogen did not cause significant decrease of red clover root length, but did cause a very strong increase of wheat root length. Significant inhibition of red clover root length was only observed in soil samples spiked with the highest concentration mixture of explosives; low concentrations caused wheat growth stimulation. Length of roots in 1,000 mg/kg concentration were significantly lower in comparison with the control samples (Fig. 2).

Among the analyzed compounds, only TNT had a significant effect on seed germination. In highest concentration, only about 30 % of the red clover seeds germinated.

Some results obtained in phytotoxicity tests are in agreement with other researchers’ achievements. The increase of toxic effects observed with the increase of TNT concentration in soil has been reported in many papers (Krishnan et al. Krishnan et al. 2000b; Vila et al. 2008). The scale of the toxic effects depended on the plant species. Wheat biomass loss was significant in comparison with control samples only at the lowest concentrations; for clover, it was at the concentration of 180 mg/kg. At the same time, 2,4,6-trinitrotoluene did not have an adverse effect on wheat germination in all analyzed concentrations, while in samples with TNT concentrations of 540 and 1,000 mg/kg only about 30 % of red clover seeds germinated. Previous research has shown that a soil’s sensitivity to the presence of TNT depends on plant species. Alfalfa (*Medicago sativa*) could not grow in soil contaminated with TNT at a concentration of 100 mg/kg (Scheidemann et al. 1998). Similarly, cress (*Lepidium sativum*) and cabbage (*Brassica rapa*) germination was inhibited at the TNT concentration of 200 mg/kg in the soil (Gong et al. 1999). Oats (*Avena sativa*) demonstrates significant resistance to the presence of trinitrotoluene in soil, for which no toxic effects were observed even at a concentration of 1,600 mg/kg (Gong et al. 1999). RDX did not affect the germination of red clover and wheat. These observations are similar to the results obtained by Best et al. (2006), who conducted tests with ryegrass (*Lolium perenne*) and alfalfa (*Medicago sativa*), and by Winfield et al. (2004), who analyzed the effects of hexogen on 16 different plants.

In this research, red clover and wheat biomass loss and morphological changes did not appear in plants grown in the soil spiked with hexogen. In soil contaminated with RDX (138 mg/kg concentration), Vila et al. (2007b) observed necrosis and bleaching on the surfaces of leaves of wheat (*Triticum aestivum*), rice (*Oryza sativa*), and soybean (*Glycine max*). Alternatively, in research conducted by Rocheleau et al. (2008) no adverse effects in ryegrass (*Lolium perenne*) were observed at RDX concentrations up to 10,000 mg/kg. Differences between obtained results may be connected with different test durations: toxic effects appeared in tests conducted for 42 days (Vila et al. 2007b). Presumably, 14 days (this research) or 21 days (Rocheleau et al. 2008) may be a too short period to notice in plants the appearance of morphological changes caused by RDX exposure. Despite the fact that during the tests no adverse changes in wheat were observed, the effects of increased growth are difficult to predict. Tests of greater duration would provide a response if any changes were to appear. The presence of octogen in soil did not cause adverse effects on wheat and red clover germination and growth (it caused only growth stimulation). This is similar to research conducted by Rocheleau et al. (2008), in which lettuce (*Lactuca sativa*), barley (*Hordeum vulgare*), and ryegrass (*Lolium perenne*) tolerated very high HMX concentrations in artificial soil (lettuce and barley to 3,320 mg/kg; ryegrass up to 10,000 mg/kg).

In the evaluation of zootoxicity, the impact of explosives contamination in soil on earthworm (*Eisenia fetida*) mortality was determined. The highest earthworm mortality was observed in soil spiked with TNT. In samples where TNT concentration was 360 mg/kg oligochaete lethality was 70 %; in the higher concentrations, 100 %. In samples spiked with RDX, mortality was 60 % in concentrations of 540 mg/kg and LC_50_ was evaluated at the level of 585.7 mg/kg. Among the analyzed compounds, HMX showed the lowest level of earthworm toxicity. Lethality at the level of 60 % appeared only at the highest concentration (1,000 mg/kg) and LC_50_ was 841.5 mg/kg. In samples with the mixture of explosives, 100 % mortality appeared at the concentrations of 180 mg/kg and higher. Interestingly, at the lowest concentration (100 mg/kg) all the earthworms stayed alive. The obtained results indicate that in all probability it was the synergistic effect of explosives on oligochaetes that was observed. In the sample where the concentration of sum of the explosives was 180 mg/kg (60 mg TNT + 60 mg RDX + 60 mg HMX), complete mortality was observed; in the tests with individual explosives, it was only higher concentrations that caused a lethal effect. The test was repeated with the lower concentrations (100, 130, 170, 220, 290 mg/kg) to more precisely observe mortality changes. The effects were surprising: again at 100 mg/kg almost all earthworms stayed alive (39 out of 40), while in the next concentration – 130 mg/kg – only one living organism was observed after 7 days incubation time. LC_50_ was evaluated at the level of 115 mg/kg. After calculating toxicity units (TUs), which are defined as 100 divided by the EC_50_ or LC_50_ (Kalka 2012), the synergistic effect of the mixture of explosives in comparison with individual substances tests was assessed. The result was positive (Table 2), which is proof of the synergistic effect of explosives in a mixture.

**Table 2:** Earthworm mortality results and mixture synergistic effect assessment

Explosives in soil	LC_50_ (mg/kg)	Earthworm mortality	
		TU^a^	Synergistic effect
TNT	276.7	0.36	
RDX	585.7	0.17	
HMX	841.5	0.12	
MIX (TNT + RDX + HMX)	115.0	0.87	Positive (TU_MIX_^b^ > TU_X_^c^)

^a^ *TU* toxic unit (100/LC_50_)

^b^ *TU*_*MIX*_ mixture toxic unit (0.87)

^c^ *TU*_*X*_ the sum of TNT, RDX and HMX individual TUs (0.65)

TNT zootoxicity tests results are in agreement with other researchers reports. A 50 % mortality rate of the population of the earthworm (*Eisenia fetida*) was estimated at the concentration level of 276.7 mg/kg. According to other reports, LC_50_ evaluated for different forest soils was 143–325 mg/kg (Lachance et al. 2004). Results obtained in artificial soil are considerably higher, which indicates that the scale of the toxic effect depends on the soil content (Lachance et al. 2004). It has been stated in many investigations that RDX and HMX do not cause lethal effects, even in high concentrations (Robidoux et al. 2002; Best et al. 2006). In this research, earthworm mortality was observed in soil spiked with hexogen and octogen. These compounds were lethal for oligochaetes at higher concentrations than TNT: the LC_50_ value estimated for RDX was 585.7 mg/kg and for HMX it was 841.5 mg/kg. This unexpected effect can be connected with physical–chemical properties of soil and soil content, especially with an organic matter content. In the soil with a low organic matter content the small amount of explosives is binding to soil matter. Almost all introduced substances are bioavailable to organisms. It is also the first time that the synergistic toxic effect of the mixture of TNT, RDX, and HMX on earthworm survival has been observed. The concentration of explosives that caused lethality of *Eisenia fetida* was lower in the samples with explosives mixture than in the samples with individual substances.

The analyzed compounds are toxic, and the scale of the effects they cause is difficult to predict. In comparing results obtained in this research with data reported by other researchers, it can be stated that toxic effects depend not only on the concentration of explosives, but also on the kind of soil in which they appear. Furthermore, mixtures of explosives can cause other and/or stronger effects than individual substances. That is why there is a need to conduct more research to assess the effects of different explosives compositions on various organisms.
